# Physiology of Weight Regain after Weight Loss: Latest Insights

**DOI:** 10.1007/s13679-025-00619-x

**Published:** 2025-03-31

**Authors:** Marleen A. van Baak, Edwin C. M. Mariman

**Affiliations:** 1 Department of Human Biology, NUTRIM Institute of Nutrition and Translational Research in Metabolism, Faculty of Health, Medicine and life Sciences+, Maastricht University, Maastricht, The Netherlands; 2 Department of Human Biology, NUTRIM Institute of Nutrition and Translational Research in Metabolism, Maastricht University, PO Box 616, Maastricht, 6200MD The Netherlands

**Keywords:** Obesity, Lean mass, Immune memory, Gut microbiota, Weight regain

## Abstract

**Purpose of Review:**

This review summarizes the most recent research on the physiology of weight regain. It describes developments in areas that are currently being addressed and that may indicate promising directions for future research.

**Recent Findings:**

Weight regain occurs independent of the way prior weight loss is achieved, i.e. by lifestyle, surgery or pharmacotherapy. Recent novel findings regarding weight regain belong to four areas. First, the immune obesity memory of which besides persistent immune cells promoting weight regain cells have been found that reduce weight regain. Second, the gut microbiome where autologous transplantation can limit weight regain. Third, the composition of the weight loss with the percentage of lost fat-free mass being inverse to the amount of regained weight independent of the weight loss procedure. Fourth, appetite control where after weight loss altered hypothalamic activity promoting hunger and weight regain persists, possibly mediated by altered neurotensin responses. In all four areas more conclusive evidence for their role in weight regain still needs to be obtained.

**Summary:**

Most studies on physiological mechanisms of weight regain are associative in nature and the number of intervention studies is very limited. To bring the field further, carefully designed intervention studies taking into account the dynamic character of weight loss and weight regain are needed.

## Introduction

**Fig. 1:**
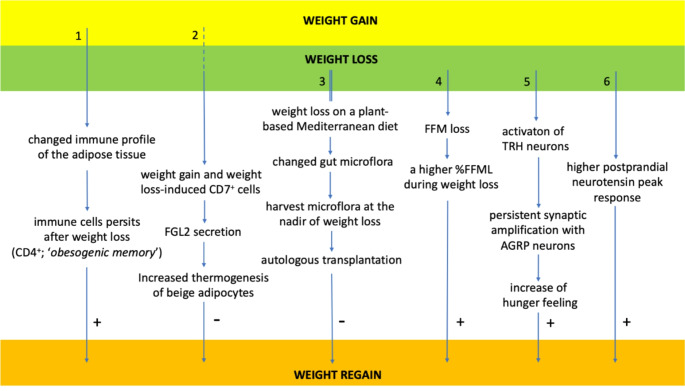
Novel findings related to weight gain- and weight loss-induced processes that may influence the risk of weight regain discussed in this review. Four physiologic areas are concerned: [1,2] the immune cell profile of adipose tissue, [3] the gut microbiome, [4] fat free mass loss during weight loss, and [5,6] appetite control. It should be noted that all these influences require further investigation to establish whether they play a causal role in the process of weight regain

Based on the most recent report of the NCD Risk Factor Collaboration [1], it is estimated that more than one third of the world population was overweight or obese in 2022 [2]. Because overweight and obesity are associated with increased health risk, effective interventions to lose weight by lifestyle changes, pharmacotherapy or bariatric surgery have been developed. However, weight regain after successful weight loss is frequent [3]. Therefore, combatting weight regain after successful weight loss is one of the major challenges of obesity management. Knowledge about the mechanisms leading to weight regain is needed in order to develop strategies to prevent weight regain. Over the past five years a number of reviews and reports from symposia have focused on the physiology of the weight-reduced state and physiological mechanisms of weight regain [4–9]. They concluded that the weight-reduced state differs from the non-obese state and that the mechanisms playing a role in weight regain after weight loss are likely different from those playing a role in initial weight gain. Potential weight regain mechanisms addressed in these reviews are: weight loss-induced variations in cellular stress and extracellular matrix remodelling of adipocytes, immune cell profile and inflammatory responses in adipose tissue, adipokine secretion, lipolysis and lipid oxidation, energy expenditure and metabolic adaptation, appetite-related hormones and the gut-brain axis, and epigenetic modifications, including regulation by microRNAs. In this review we will give an update of new developments on some of these topics, mainly focusing on the literature of the last three years. For this narrative review, we searched the literature (PubMed) for studies on weight regain or weight (loss) maintenance and selected those that focused on the potential physiological mechanisms. These are summarized in Fig. 1. A short description of the human intervention studies included in this review can be found in Table 1.

**Table 1:** Characteristics of the experimental studies in humans included in the review

Author (year)(ref)	Study design	Population	Findings	Comments
Brethvad et al. (2023) [43]	RCT: 8-week LCD followed by 1 year WM program with or without liraglutide	42 participants with obesity	13% reduction in BW associated with 40% reduction of plasma neurotensin. No difference in WM between groups. Meal-induced peak neurotensin after WL higher in those that lost additional weight during follow-up than in weight regainers	causality not demonstrated
Corbin et al. (2023) [33]	RCT: 19 d of 1000 kcal reduced diet with or without a GLP1/glucagonRA	35 participants with overweight or obesity	drug-treated group had smaller reduction in body composition adjusted SMR compared to placebo	weight regain not studied
Dubin et al. (2024) [36]	analysis of 28 WL interventions with GLP1-RAs using DXA to provide references for expected changes in FFM	28 clinical trials	%FFML ranged between 20 and 40%; highly variable, but mostly > 25%	
Faria et al. (2020) [26]	Cross-sectional study: gut microbiota at least 5 years after RYGB surgery	34 women with morbid obesity	significant difference in gut microbiota composition between WM and WR	causality not demonstrated
Hinte et al. (2024) [20]	longitudinal intervention study: single-nucleus RNA sequencing in sc and omental AT before and after bariatric surgery	20 individuals with obesity and > 25% BW loss 2y post-surgery	retained transcriptional differences in AT cells after significant WL that normalized BW	WR not studied
Jensen et al. (2024) [13]	RCT: 8-wk LCD followed by 52 weeks RCT (exercise, liraglutide, exercise plus liraglutide or placebo) and one-year follow-up	109 participants	During follow-up (week 52–104), WR was 6.0 kg (2.1; 10.0) larger after termination of liraglutide compared with supervised exercise and 2.5 kg (− 1.5 to 6.5) compared with combination treatment	Same study as Lundgren et al.
Kamer et al. (2023) [28]	RCT: aFMT or placebo over 6 months after initial WL by 6 months lifestyle intervention with 3 different diets	90 overweight, sedentary participants with > 3.5% WL during lifestyle intervention	participants with a low 6 month change in core taxa, and a high change in non-core taxa, avoided 8–14 month WR with aFMT treatment	Same study as Rinott et al.
Look et al. (2025) [39]	substudy of SURMOUNT-1 RCT in centers that used DXA to measure body composition; tirzepatide or placebo treatment for 72 weeks	160 participants without T2DM with BMI ≥ 30 kg/m^2^ or ≥ 27 kg/m^2^ with one or more weight-related co-morbidities	approximately 25% of BW loss was FFM with no difference between tirzepatide and placebo	
Lundgren et al. (2021) [40]	RCT: 8-wk LCD followed by 1 year RCT: placebo, exercise, liraglutide or combination	195 participants with obesity	exercise training resulted in a more favourable change in body composition (more increase in lean mass) associated with weight regain than liraglutide treatment	
Martins et al. (2023) [31]	longitudinal intervention study: 8 wk VLCD, 4 wk refeeding and weight stabilization, 1 year follow-up	70 participants with obesity	%FFML at week 9 was not a significant predictor of WR	
Rinott et al. (2021) [27]	RCT: aFMT or placebo over 6 months after initial WL by 6 months lifestyle intervention with 3 different diets	90 overweight, sedentary participants with > 3.5% WL during lifestyle intervention	aFMT treatment reduced WR in the green Mediterranean (polyphenol-rich) diet group only	
Spranger et al. (2023) [32]	RCT: 3 month dietary WL intervention followed by 4 weeks weight stability and 20 months follow-up without active intervention	80 post-menopausal women with overweight or obesity	a lower RMR per kg FFM after WL and less increase in this parameter during the 4-wk period of weight stability were associated with more regain of FM during follow-up	causality not demonstrated
Turicchi et al. (2020) [30]	substudy of DIOGENES trial RCT; 8 wk VLCD followed by RCT with 5 different dietary advices	209 participants with overweight or obesity	%FFML after 8 wk tended to be associated with WR after 1 year (*P* = 0.055)	causality not demonstrated
Vink et al. (2016) [29]	RCT with longitudinal follow-up: 5–12 wk WL with VLCD or LCD, 4 wk weight stabilization, 9 months follow-up	57 participants with overweight or obesity	%FFML from wk 0 to wk 9–16 associated with WR	causality not demonstrated
Weintraub et al. (2023) [14]	Longtitudinal cohort study: duration 2.5-5 y	428 patients with overweight or obesity treated with AOM	51% of maximum WL was regained, 40% of patients maintained their WL	
Wilding et al. (2022) [12]	STEP1 RCT: 68 weeks semaglutide or placebo followed by 1 year off-treatment extension	327 participants of the STEP1 trial without T2DM with BMI ≥ 30 kg/m2 or ≥ 27 kg/m2 with one or more weight-related co-morbidities	Following treatment withdrawal, semaglutide and placebo participants regained 11.6 (SD: 7.7) and 1.9 (SD: 4.8) percentage points of lost weight, respectively, by week 120, resulting in net losses of 5.6% (SD: 8.9%) and 0.1% (SD: 5.8%), respectively, from week 0 to week 120	

Abbreviations: RCT: randomized controlled trial; LCD: low-calorie diet; WM: weight maintenance/maintainers; BW: body weight; WL: weight loss; GLP1RA: glucagon-like peptide 1 receptor agonist; SMR: sleeping metabolic rate; DXA: dual energy X-ray absorptiometry; FFM: fatfree mass; %FFML: FFM loss as proportion of WL; RYGB: Roux-en-Y gastric bypass; WR: weight regain(ers); AT: adipose tissue; aFMT: autologous fecal microbiome transplantation; T2DM: type 2 diabetes mellitus; BMI: body mass index; VLCD: very low caloriediet; RMR: resting metabolic rate; AOM: anti-obesity medication

## Weight Regain after Successful Weight Loss

Weight regain after weight loss occurs independent of the way the weight loss was achieved. It has long been known that weight regain occurs after lifestyle interventions [10]. This could be due to reduced adherence to the necessary lifestyle changes, but weight regain also takes place after bariatric surgery [11]. Recently, more long-term studies have become available on weight regain after stopping pharmacotherapy. In a study by Wilding et al. (2022) [12] persons with obesity treated with the GLP1 receptor agonist semaglutide for 68 weeks lost 17% of their body weight. At week 68 treatment was discontinued. One year later they had regained about 2/3 of the lost weight. Jensen et al. (2024) [13] reported the weight regain after stopping liraglutide pharmacotherapy, another GLP1 receptor agonist. One year after stopping liraglutide, body weight had increased and was no longer different from body weight in the placebo-treated group.

Weight regain with continued pharmacotherapy also occurs. Weintraub et al. (2023) [14] showed a weight regain of 6.5% (SD 7.4%) compared to the nadir of weight loss after 2.5 to 5.5 yrs of pharmacotherapy with FDA-approved and off-label medications. Weight regain was found in half of the patients. No such longer-term data are yet available for continued treatment with the newer anti-obesity medications.

Thus, weight regain occurs no matter how the weight loss is achieved, by lifestyle changes including behavioral therapy, bariatric surgery or pharmacotherapy. Rosenbaum and Foster (2023) [7] argue that in most studies weight loss occurs over the first 6–9 months of treatment and then weight regain sets in with a rate that is remarkably similar with all treatments as far as is currently known. This may suggest common mechanisms for weight regain induced by prior weight gain and/or loss. Recent insights in some of the potentially involved physiological mechanisms will be reviewed below.

### Immune Obesity Memory

When obesity develops the number of immune cells in the adipose tissue increases and they acquire more pro-inflammatory characteristics. This often results in a low grade inflammation of the adipose tissue and a low level of systemic inflammation. Studies in mice have shown that part of the changes that occur in the immune system of the adipose tissue during obesity persist after weight loss by caloric restriction [15]. It was proposed that the presence of (some of) these immune cells after weight loss keeps the adipose tissue in an obesity-prone state, serving as an immune-based ‘obesity memory’ that underlies the risk of weight regain. Evidence for involvement of CD4^+^ T cells in the obesity memory in experimental animals has been provided [16]. CD4^+^ T cells sustain weight regain after weight loss. Recently, it was shown that induced immune cells can also have the opposite effect, i.e. the reduction of the risk of weight regain. CD7^+^ monocytes isolated from the bone marrow of mice that went through a weight gain/weight loss cycle, referred to as ‘nutritional stress-activated CD7^+^ monocytes’, were found to act against weight gain in mice on a HF diet [17]. Depletion of CD7^+^ cells from mice on a weight-cycle diet led to increased weight regain. Transferring CD7^+^ monocytes into these mice decreased body weight gain compared to transfer of CD7^−^ monocytes [17]. Apparently, obesity and weight loss modify the immune system of the adipose tissue in a way that results in opposing immunological influences on weight regain. Not much is known about possible mechanisms, but the nutritional stress-activated CD7^+^ cells secrete fibrinogen-like protein 2 (FGL2) that interacts with transmembrane protein 120 A (TMEM120A), a receptor on beige adipocytes. This promotes beige thermogenesis, explaining the attenuation of weight regain. It was further observed that with increasing time after weight loss the number of active CD7^+^ cells declines. As the CD7^+^ cells enter into a quiescent state, the inhibiting effect on weight regain becomes less [17]. Interestingly, weight regain could be limited by treating mice with the receptor tyrosine kinase FLT3, a bone marrow factor involved in hematopoietic development, which increased the number of active CD7^+^ cells [17]. It shows that manipulating the immune system in the proper way could limit the risk of weight regain.

Similar processes could be involved in humans. It has been reported that persons with obesity have a significantly higher percentage of peripheral CD7^+^ monocytes than lean individuals [18]. The percentage of circulating CD7^+^ monocytes is negatively associated with the degree of weight regain 6 months after weight loss [17]. Compared to men with normal weight or with obesity, men with at least 10% weight loss have a significantly higher CD7-gene expression in immune cells of the bone marrow, and they have also a significantly higher number of peripheral CD7^+^ monocytes [17]. It suggests that both obesity and weight loss contribute to the CD7^+^ propagation. In the mouse, nutritional stress-activated CD7^+^ monocytes are formed in the bone marrow and migrate to the inguinal white adipose tissue [17]. Since hematopoietic differentiation is influenced by epigenetic modulation of transcription factors [19], it suggests that epigenetic changes in the bone marrow induced by obesity and weight loss contribute to the generation of the active CD7^+^ monocytes. The obesity memory in general seems not only to be the consequence of complex epigenetic changes in the bone marrow, but also in the adipose tissue. Hinte et al. [20] showed, by means of single nucleus sequencing of adipocytes, that a considerable percentage of genes with obesity-induced changes of expression remained at the altered level of expression two years after bariatric surgery with 25% or higher loss of body weight. Similar observations on gene expression were made in adipocytes of mice, when mice that had gained weight on a 12 week high-fat diet followed by weight loss on a chow diet were compared to mice of similar body weight that were maintained on the chow diet for the whole period [20]. Of the adipocyte genes with persistent changes in translational activity after weight loss, about 60–75% of the persistent changes could be explained by epigenetic mechanisms like histone modifications and chromatin accessibility, arguing for a role of adipocyte epigenetics in the obesity memory.

## Gut Microflora

Since it became possible to study the composition and dynamics of the gut microflora via metagenome sequencing, the characteristics of the microflora of persons with obesity and its influence on health parameters have been investigated. In their review Liu et al. (2021) [21] reported that obesity is associated with defined changes of the microflora and that changes in the relative abundance of specific microbiota are related to processes involved in weight regulation. This includes energy supply to the host, energy absorption, appetite, fat storage, chronic inflammation, and the circadian rhythm. Transplantation experiments in mice indicate that the gut micobiome has the capacity to alter the risk of obesity and fat storage [22, 23]. However, after a critical review of studies on the relation between the microbiome and obesity, Dalby warns that, as yet, a firm conclusion on a causal role of the microbiome in obesity cannot be drawn [24].

Nevertheless, an interaction between microbiome composition and the risk of weight regain after weight loss seems likely. Chen et al. (2022) [25] showed that normal weight mice on a 4-week 30% calorie restricted diet adjusted their microbiome in such a way that it reduced weight regain on a subsequent high fat diet. A study among women with morbid obesity, studied 5 years after Roux-en-Y gastric bypass surgery, showed a considerable difference between the gut microbiome of the women who regained weight and those who maintained the lost weight. Several genera were found to differ, including *Akkermansia*, *Phascolarctobacterium*, and *SMB53* [26].

Transplantation of the microbiome has been explored as a means to reduce the risk of weight regain after weight loss in persons with obesity. So far, positive results have been obtained by autologous transplantation of faecal microbiota harvested from an individual at the nadir of a weight loss intervention (6 months) by calorie restriction and exercise, and administered at regular times 6–14 months after weight loss [27]. A clear influence of the composition of the weight loss diet was reported. Notably, the weight regain-lowering effect was only observed in a group of participants who lost weight on a plant-based Mediterranean diet that included the consumption of 3–4 cups/d of green tea and a green shake of 100 g/d Wolffia globosa duckweed (Mankai duckweed). It was suggested that components of this diet such as the high polyphenol content, support the preservation of specific weight-loss associated bacteria and microbial pathways. A further analysis revealed that the attenuation of weight regain was associated with a low change of abundant gut microbiome taxa and high change of non-abundant taxa [28]. Additional experiments are needed to assess the power of microbiome manipulation for preventing weight regain after weight loss.

## Fat-free Mass Loss and Metabolic Adaptation

The composition of the weight lost with obesity treatment has been suggested to play a role in weight regain, with a larger proportion of calorie restriction-induced weight loss due to loss of fatfree mass (%FFML) being associated with more weight regain [29, 30]. A larger loss of fatfree mass could be expected to reduce energy expenditure and trigger increased appetite, thus leading to weight regain. However, associations between %FFML and change in appetite perceptions during weight loss were inconsistent in the study by Turicchi et al. [30]. In addition, a recent study could not confirm the relationship between %FFML and weight regain 1 year later, although a greater %FFML was accompanied by a greater increase in ghrelin secretion under ketogenic conditions, suggesting a link between fat-free mass and appetite regulation [31]. Inconsistencies may be related to the conditions (e.g. energy balance and stable body weight or not, time after weight loss) under which both FFM loss and appetite parameters were measured.

A recent study by Spranger et al. (2023) [32] found no association between the loss of fatfree mass induced by calorie restriction and regain of fat mass 20 months later. However, a lower resting metabolic rate per kg fatfree mass after weight loss and less increase in this parameter during the 4-wk period of weight stability after the weight loss intervention, suggestive of a thrifty phenotype, were associated with more regain of fat mass during the 20-month follow-up period. The investigators identified the weight loss-induced adaptation of adipose tissue fibroblast growth factor receptor 1 (FGFR1) signaling as a potential mechanism underlying the individual adaptation of resting metabolic rate to a negative energy balance. However, a functional analysis of its role in weight regain is so far lacking. Interestingly, in a phase 1B trial with a novel GLP1/glucagon receptor agonist, it was found that the weight-loss induced reduction in sleeping metabolic rate adjusted for changes in body composition was smaller in the agonist-treated group than in the placebo group [33]. Such blunted metabolic adaptation may be important for weight loss maintenance [34], but its relevance awaits further testing.

In 2024, several reviews have addressed the importance of the composition of weight loss in the context of the large fatfree mass losses seen with bariatric surgery and the newer anti-obesity medications [35–38]. Argyrakopolou et al. (2024) [35] concluded, based on a limited number of studies, that drugs such as liraglutide, semaglutide, tirzepatide and naltrexone/bupropion may have a positive effect on body composition, i.e. cause relatively little loss of lean body mass. Dubin et al. (2024) [36] reviewed the weight loss composition as measured with dual energy X-ray absorptiometry (DXA) or air displacement plethysmography (ADP) with glucagon-like peptide-1 receptor agonist (GLP1RA)-based agents, such as luraglutide, semaglutide, exenatide and tirzepatide. DXA and ADP measure fatfree mass, approximately half of which is lean body mass. Based on the results of 28 clinical trials they found that %FFML ranged between 20% and 40%, with most studies reporting a %FFML above 25%. A recent analysis of participants of the SURMOUNT-1 trial in whom body composition was measured by DXA found that %FFML after 72 weeks of treatment was 25% and similar in the tirzepatide-treated groups (all doses combined) and the placebo-treated group [39]. Together with the data in the review by Dubin et al. [36], this may suggest that tirzepatide, an agonist of GLP1R as well as GIP (glucose-dependent insulinotropic polypeptide) receptor, has a more favourable impact on body composition changes than other GLP1RA-based drugs. Stefanakis et al. (2024) [37] reported similar %FFM losses for pharmacotherapy with incretin-mimetics and bariatric surgery. In contrast, most dietary interventions are associated with %FFML < 25% [36]. Mechanick et al. (2024) [38] report that clinical trial participants receiving incretin-mimetic drugs lost more than 10% of muscle mass over 68 to 72 weeks.

A study by Lundgren and coworkers (2021) [40] showed that exercise training after diet-induced weight loss resulted in a more favourable change in body composition (more increase in lean mass) associated with weight regain during follow-up than liraglutide treatment. Although more studies are needed to confirm the role of %FFM or muscle mass loss for weight regain, it seems prudent to try to limit lean mass loss during obesity treatment, for instance by regular (resistance) exercise supported by a diet with beneficial protein content and composition. Currently drugs that increase skeletal muscle by interfering with the myostatin pathway are being investigated for obesity treatment, in combination with GLP1RA-based drugs to preserve lean mass [37, 41].

## Appetite Control

Most of the mechanisms involved in weight regain, including those described above, will indirectly influence appetite control. However, only few studies have provided information on a more direct role of appetite-control mechanisms in weight regain. Gerstenberg et al. [42] analysed the weight loss-induced changes in anorexigenic hormones secreted into the lumen of the small intestine under fasting conditions and after a meal in diet-induced obesity (DIO) rats. Fasting or postprandial secretion of cholecystokinin, gastrin, glucose-dependent insulinotropic peptide, GLP1, neurotensin, and somatostatin was not affected by weight loss. In contrast, Brethvad et al. [43] studied the role of the gut-derived anorexigenic hormone neurotensin in weight maintenance in mice and humans. In mice, plasma concentrations of neurotensin were reduced after weight loss by food restriction. In the hypothalamus *Pomc* expression was downregulated and *NPY* and *Agrp* expressions were upregulated, suggestive of increased hunger after weight loss. In humans with obesity, weight loss induced by calorie restriction also reduced fasting neurotensin concentrations. However, after weight loss individuals who subsequently regained weight had higher peak neurotensin responses after a mixed meal than individuals who continued to lose some weight over the 1-year weight maintenance period [43].

A study in mice by Grzelka et al. [44] looked at calorie restriction-induced changes in the hypothalamus. After weight loss TRH-neurons of the paraventricular hypothalamus were activated and caused a persistent synaptic amplification with AgRP neurons, leading to increased hunger and weight regain.

So far, these studies seem to support the concept that weight loss by calorie restriction induces an adjusted hypothalamic activity which persists afterthge calorie restriction, perhaps mediated by altered gut hormone responses, and which increases hunger and the risk of weight regain. However, more detailed studies are needed to further investigate this concept.

## Perspectives

In the previous decade significant progress has been made on the control of weight gain leading to overweight and obesity and on procedures of subsequent weight loss. However, to maintain the lost weight appears difficult with weight regain being very common. Methods used to control weight gain may not be adequate to reduce the risk of weight regain, because the mechanisms causing weight regain seem to differ from those of the development of the initial weight gain. It means that we are facing a profound challenge to obtain more insight in how weight regain develops. As is obvious from the present and previous reviews, so far only a limited number of studies have been performed into the background of weight regain and knowledge regarding the underlying mechanisms is greatly lacking. Moreover, most of these studies are associative, whereas unravelling the mechanisms of weight regain requires a more systematic approach in which cellular and molecular clues as well as associative findings are tested in human intervention studies. Results of such studies will have to be combined and analysed in a personalized manner, which requires modern methods such as complex data analytics and artificial intelligence. Initiating these studies and analytical approaches is the main research goal of the coming years to eventually succeed in controlling the obesity pandemic.

## Conclusions

Weight regain after successful weight loss has gradually become acknowledged as one of the major challenges for long-term obesity management. Weight regain occurs frequently, independent of the way weight loss is achieved. i.e. by lifestyle intervention, bariatric surgery or pharmacotherapy. Recent research on the physiological mechanisms underlying weight regain identified a role for epigenetic changes in the bone marrow and adipocytes. Obesity- and weight loss-induced differentiation of immune cells in the bone marrow leads to cells that sustain weight regain as well as cells that protect against it. Another proposed mechanism is loss of lean body mass resulting in reduced energy expenditure and increased energy intake. Recent studies discussed the importance of the composition of body mass reduction and the role of large reductions of lean body mass with the currently available incretin-based pharmacotherapy in weight regain. Whether obesity-induced or weight loss-induced changes in the gut microbiota composition are causally associated with weight regain remains to be demonstrated, but the gut microbiota composition may affect energy intake and storage. Autologous transfer of microbiota collected after a weight loss intervention has been found to reduce weight regain during follow-up dependent on the composition of the weight loss diet in one study, which needs replication. Weight loss induces secretion of gut hormones like neurotensin, and adjusts signalling activities in the hypothalamus increasing hunger, which could persist after weight loss and increase the risk of weight regain. However, these observations are preliminary as only few studies have been performed and most of them in laboratory animals.

Most of the studies available on the topic of weight regain are associative in nature and there is still a lack of intervention studies that show whether interfering with the proposed mechanisms also leads to reduced weight regain. Because the cycle of weight loss and weight regain is a dynamic process, future studies should be carefully designed to separate the effects of a negative energy balance and of reduced weight, and to reflect the time course of the changes in the underlying physiology.

## Key References

- Rosenbaum M, Foster G. Differential mechanisms affecting weight loss and weight loss maintenance. Nat Metab. 2023;5(8):1266-74.
  - Explores weight regain trajectories after weight loss achieved by lifestyle, pharmacotherapy and bariatric surgery.

- Zhou HY, Feng X, Wang LW, Zhou R, Sun H, Chen X, et al. Bone marrow immune cells respond to fluctuating nutritional stress to constrain weight regain. Cell Metab. 2023;35(11):1915-30 e8.
  - Reports the discovery of weight-cycling induced immune cells that protect against weight regain. It shows that the risk of weight regain is modified by opposing immunological influences.

- Hinte LC, Castellano-Castillo D, Ghosh A, Melrose K, Gasser E, Noe F, et al. Adipose tissue retains an epigenetic memory of obesity after weight loss. Nature. 2024;636(8042):457 − 65.
  - Demonstrates that in adipocytes of WAT gene expression changes induced by weight gain persist after weight loss, and that for the genes with deregulated translation these expression changes relate to epigenetic modalities like specific histon modifications and chromatin accessability.

- Rinott E, Youngster I, Yaskolka Meir A, Tsaban G, Zelicha H, Kaplan A, et al. Effects of Diet-Modulated Autologous Fecal Microbiota Transplantation on Weight Regain. Gastroenterology. 2021;160(1):158 − 73 e10.
  - Reveals that it is possible to reduce the risk of weight regain by employing a'prebiotics to probiotics' model. It is demonstrated by weight loss on a specific green-based diet followed by ingestion of autologous fecal microbiota harvested at the nadir of weight loss.

## Data Availability

No datasets were generated or analysed during the current study.
